# Studying Intense Pulsed Light Method Along With Corticosteroid Injection in Treating Keloid Scars

**DOI:** 10.5812/ircmj.12464

**Published:** 2014-02-05

**Authors:** Simin Shamsi Meymandi, Azadeh Rezazadeh, Ali Ekhlasi

**Affiliations:** 1 Department of Dermatology, Kerman University of Medical Sciences, Kerman, IR Iran

**Keywords:** IPL, Keloid, Hypertrophic

## Abstract

**Background:**

Results of various studies suggest that the hypertrophic and keloid scars are highly prevalent in the general population and are irritating both physically and mentally.

**Objective:**

Considering the variety of existing therapies, intense pulsed light (IPL) method along with corticosteroid injection was evaluated in treating these scars.

**Materials and Methods:**

86 subjects were included in this clinical trial. Eight sessions of therapeutic intervention were done with IPL along with corticosteroid intralesional injection using 450 to 1200 NM filter, Fluence 30-40 J/cm2, pulse duration of 2.1-10 ms and palsed delay 10-40 ms with an interval of three weeks. To specify the recovery consequences and complication rate and to determine features of the lesion, the criteria specified in the study of Eroll and Vancouver scar scale were used.

**Results:**

The level of clinical improvement, color improvement and scar height was 89.1%, 88.8% and 89.1% respectively. The incidence of complications (1 telangiectasia case, 7 hyperpigmentation cases and 2 atrophy cases) following treatment with IPL was 11.6%. Moreover, the participants’ satisfaction with IPL method was 88.8%.

**Conclusions:**

This study revealed that a combined therapy (intralesional corticosteroid injection + IPL) increases the recovery level of hypertrophic and keloid scars. It was also demonstrated that this method had no significant side effect and patients were highly satisfied with this method.

## 1. Background

Hypertrophic and keloid scars occur as a result of an accelerated recovery reaction to damages to skin-deep tissues; it has a long phase of inflammation and fibroplasia. This reaction results in extensive fibroblast proliferation and collagen synthesis and finally leads to fibrosis in hypertrophic and keloid scars (1). Keloid and hypertrophic scars are wounds with abnormal appearance and both form from proliferation of local fibroblasts and increased collagen production in response to skin injury. Although these scars are more likely to occur on areas which are under pressure, some low-pressure parts such as ear lobules are prone to scars (2). Factors that play an important role in the formation of keloid include genetics, individual talents, and both of these factors plus injury and inflammation of the skin (lacerations, burns, surgery, vaccination and tattoos, acne vulgaris and bites) (3). Risk of deep burns in the creation of keloid scars is high (4). It has been proposed that hormones are likely to form keloids and that keloids can appear at the time or after puberty and can get better after menopause (5).

Prevalence of hypertrophic scars in the general population is estimated to be about 1.5 to 4.5 % (6). There is no single way to treat these scars. Scars can be surgically removed and the skin can then be sutured; other therapeutic methods including silicon coating and compression of the lesion area to reduce the lesion size, interferon, bleomycin and 5-fluorouracil (5-fu) intralesional injection and corticosteroid intralesional injection can also be used (1, 7, 8). Another method used to treat scars is VL/PL photoderm (IPL), which is a Flash lamp intense pulsed light source and emits broad wavelengths (400-1200 nm). There are numerous blocking filters in wavelengths of 515, 550, 570, 590, 615, 645 and 755 nm to prevent emission of optical ray with shorter wavelengths; thus, IPL can, by affecting melanin on scar pigmentation, be effective and recover hyperpigmented scars. In addition, it can affect scar vascular structures and help hypertrophic and keloid scares. Therefore, IPL is an effective method in treating scars (9). The aim of IPL is vascular proliferation in the field of collagen high growth on pigmentation, i.e. factors which play a role in creating hyperpigmentation, eritemato and hypertrophic scars (10). IPL can effect pigmentation of scars, vascularity of scars and can be used for the treatment of erythema of the scars (11).

In a study by Erol et al. in 2008, 109 patients with hypertrophic scars were treated using IPL; level of clinical recovery was 99.5% for scar appearance (1). In a study carried out by Konteos et al. in 2003, 19 patients underwent IPL method with intralesional injection; recovery level was more than 75% (9). In his study, Niessen et al. (1999) treated 15 patients with IPL. Recovery levels after the first and the second sessions were 45% and 65% respectively (5). Myers et al. in 2005 treated 107 patients using IPL and the recovery level was 55% (12). In a study by Han, clinical recovery was 100% with an average of 3.5 therapeutic sessions on 22 patients (7). Most physicians prefer IPL because it needs fewer therapeutic sessions to achieve the desirable results and because it is flexible and can, by changing its settings in its broad optical spectrum, be used for different skin therapeutic purposes (13). Moreover, treatment with IPL is not invasive, doesn’t need injection and has fewer side effects, which can be controlled by cooling the location and using anesthetic cream. Making use of special sheets, emission of light to skin around the lesion can be prevented (1, 14). For initiation of treatment, a test should be conducted and the skin reaction should be observed and the patient should be asked about any discomfort or local reaction. When treating circumscribed smaller lesions, the use of a perforated plastic shield with varying aperture sizes may be helpful (11, 15).

There is a high incidence of keloid scars among patients admitted to the dermatology clinic of Kerman Afzali Pour Hospital and dermatology clinics in Kerman, however common treatments are sometimes unsuccessful or need time, thus patients face numerous problems due to several referrals and lack of recovery.

## 2. Objectives

Therefore, since IPL has had successful results in other countries and since no similar studies have been carried out in Iran, IPL with corticosteroid intralesional injection was examined in a clinical trial study on patients admitted to this center.

## 3. Materials and Methods

This study was of clinical trial, which was carried out on patients admitted to Kerman Afzalipour Hospital (Kerman province of IR Iran). In this uncontrolled clinical trial, the efficiency of IPL plus corticosteroid intralesional injection was evaluated in treating keloid scars. Inclusion criteria were all colloid scares caused by trauma, surgery, acne and thermal or chemical burns. In case a patient experienced unacceptable complications, that patient was excluded. Pregnancy, breast feeding, intake of retinoid or photosensitizing medications, diseases or genetic conditions causing photosensitivity or tending to aggravate after light exposure are exclusion criteria for IPL treatment, but we did not have any of these exclusion criteria in our study. We identified that patients suffering from long term diabetes, hemophilia, or other coagulopathies and patients with implants in the treatment area or with a heart pacemaker should be treated with special care; for these individuals treatment was done using a 450 to 1200 NM filter, fluence 30-40 J/cm2, pulse duration 2.1-10 ms, palsed delay 10-40 ms and an interval of three weeks between each intervention. Also, a protective device attached to the laser head protected normal skin around the lesion. Meanwhile, 8 intervention sessions was allocated to every patient.

The study date was from 21^st^ April 2012 to 21^st^ April 2013. Due to the limited number of samples, no sampling was done and 86 subjects were entered in this study, therefore the sampling method was census. Also, given that there were eight therapeutic interventions with IPL for every patient and a three-week interval between every treatment period, the final sample was included 24 weeks before the end of this study. At the beginning of the study, study conditions and therapeutic methods were explained to all subjects; after obtaining informed consents, the subjects participated in the study. The samples could, in every stage of the research, leave the study. In addition, studies showed that complications of IPL with intralesional corticosteroid injection were not more than those of the conventional method (cryo). Also, in order to avoid additional costs, participants only paid the cost of the conventional method (cryo).

To collect information, a checklist including, age, sex, scar emergence time, scar age, reason of scar, history of scar treatment, scar clinical status in terms of vascularity, pigmentation, pliability and height, and clinical improvement, color improvement, scare height and patients’ satisfaction with IPL, was designed. Due to the nature of the objective variables, reliability and validity of the checklist was 100%. Moreover, to prevent sample exclusion, some guidelines were presented including appropriate counseling at the first referral session and telephone follow-ups for future referrals. To determine the expected outcomes of the study (complete recovery and poor, average, good and excellent recovery level after each intervention and level of complications) and to determine lesion features, criteria defined in the study of Eroll (1) and Vancouver scar scale (16, 17) were used (as follows).

Assessment of any scar with a determination of its extent (i.e. surface area or volume) was performed based upon measurement of the maximum dimensions and thickness. In addition, subjective scales, such as the Vancouver scar scale (VSS) (Table 1), have been developed to provide on overall impression of the quality of the scar. Noninvasive techniques can be used to measure the scar and its color, blood flow, transcutaneous oxygen, skin hardness, elasticity and hydration. However, the eventual goal was to create an easy-to-use and inexpensive system that monitors the qualitative and quantitative evolution of the scar and one that is widely accepted.

**Table 1.** Vancouver Scar Scale

Score for Each Clinical Feature	0	1	2	3	4
**Pliability**	normal	supple	yielding	firm	adherent
**Height**	normal	1-2 mm	3-4 mm	5-6 mm	> 6 mm
**Vascularity**	normal	pink	red	purple	-
**Pigmentation**	normal	hypopigmentation	hyperpigmentation	-	-

Concerning classification of treatment results, scar treatment was 25% in the “poor result group”, 26-50% in the “average result group”, 51-75% in the “good result group” and more than 75% in the “excellent result group”. For data entry and analysis, SPSS 20 software was used; data was analyzed using classic chi-square test and t-test. It should be noted that the significance level of the tests was set at 5%. This study was proposed to the ethics committee of Kerman University of Medical Science and was approved with code number 140/91.

## 4. Results

Of the 86 studied subjects, 61.6% (53 cases) were female with an average age of 18.3 ± 32.7 years. Also, the difference between the average age of males and females was not significant (P = 0.2). The majority of subjects were in the age group of 21-40 years (55.8%) and scar appearance age in most of them was between 21 and 40 years of age (55.8 %). Disease period was equal or less than 1 year for 9 cases and equal or more than 10 years for 13 cases; however, for most cases disease period was 2 years (42 cases). Concerning scar locations, they appeared on the chest in 9 cases, on epigastria and abdomen in 6 cases, on hands in 14 cases, on breasts in 7 cases, on neck in 1 case, on leg in 7 cases, on ears in 2 cases, on shoulders in 11 cases, on deltoid in 4 cases and on arms in 25 cases. Surgery was the cause of scars in 14 cases, acne in 20 cases, trauma and rupture in 34 cases, folliculitis in 2 cases and burn in 16 cases. Concerning scar clinical status in terms of vascularity, 28 cases had pink, 45 cases had red and 13 cases had purple scars. Regarding scar clinical status in terms of pigmentation, 7 cases had hypopigmentation, 37 cases had mixed and 42 cases had hyperpigmentation. Concerning scar clinical status in terms of pliability, 1 case had supple, 10 cases had yielding, 64 cases had firm, 4 cases had contracture and 7 cases had rope scars. Regarding scar clinical status in terms of height, in 16 cases scars were less than 2 mm, in 31 cases 2-5 mm and 39 cases more than 5 mm.

Level of clinical improvement, color improvement and scar height was 89.1, 88.8 and 89.1%, respectively. Improvement level was poor in 4 cases, average in 6 cases, good in 62 cases and excellent in 63 cases (73.2 %). It should be mentioned that complete recovery and improvement (100%) was observed in 62 cases (72.1%). Average improvement was 23.2% after one intervention (with one case achieving complete improvement (100%) after one intervention). Improvement level was poor (equal or less than 25%) in 69.8% of subjects after first treatment with IPL. 85 cases underwent intervention for the second time and improvement level was 42.6%; 7 cases achieved complete improvement. 78 cases participated in the third intervention whose improvement level was 55.8%; 16 case achieved full improvement. 62 cases underwent the forth intervention in which improvement increased by 60.9% (10 cases had full recovery). 52 cases accepted to participate in the fifth intervention with improvement level of 65.4%. In this group, 13 cases achieved complete recovery. Improvement level for 35 cases that participated in the sixth intervention was 67.1% with 3 cases recovering completely. Seventh intervention was done on 32 cases; average improvement was 75.4% with 8 cases having full improvement. Finally, 23 cases experienced 71.7% recovery after the eighth intervention and 4 cases had full recovery. However, improvement level in one case was still poor after the eighth intervention; in this case, the scar was on the patient’s hand and was due to burns. It is worth mentioning that 4 cases quit the study after the fifth intervention and 1 subject quit after the seventh intervention (before achieving full improvement). The incidence of complications following treatment with IPL was 11.6% (10 cases); telangiectasia in 1 case, hyperpigmentation in 7 cases and atrophy in 2 cases. Also, 88.8% of patients were satisfied with the IPL method.

Good recovery and improvement was achieved more often in men than in women and was greater in the age group of 11-20 when compared to the other age groups; these differences were statistically significant. Moreover, good recovery in patients with average disease period equal to or more than 10 years was less than those with average disease period of less than 10 years (Table 2 and 3). Table 4 shows that differences between recovery levels in terms of location were statistically significant and scars caused by burns had the lowest recovery level. According to Table 5, excellent recovery in purple scars is significantly less than that in pink and red scars (P = 0.07); excellent recovery in hyperpigmentation scars is less than Hypopigmentation and mixed scars (P = 0.06); rope scars have less recovery than supple, yielding, firm and contracture scars (P < 0.001). Moreover, excellent recovery in scars with a height equal to or less than 5 mm was significantly more than scares with a height of more than 5 mm.

**Table 2.** Describe the Distribution of the Samples by Studied Variables

Variable	No. (%)
**Gender**	-
Male	33 (38.4)
Female	53 (61.6)
**Age group**	-
≤ 10	7 (8.1)
11-20	17 (19.8)
21-40	48 (55.8)
41-60	5 (5.8)
≥ 61	9 (10.5)
**Average scar duration**	-
≤ 1	9 (10.5)
2	42 (48.8)
3	10 (11.6)
4-5	2 (2.3)
6-9	10 (11.6)
≥ 10	13 (15.1)
**Member of overtaken**	-
Chest	9 (10.5)
Epigastria and Abdomen	6 (7)
Hand	14 (16.3)
Breast	7 (8.1)
Neck	1 (1.2)
Leg	7 (8.1)
Ear	2 (2.3)
Shoulder	11 (12.8)
Deltoid	4 (4.7)
Arm	25 (29.1)
**Cause of scar**	-
Surgery	14 (16.3)
Acne	20 (23.3)
Trauma and Rupture	34 (39.5)
Folliculitis	2 (2.3)
Burn	16 (18.6)
**History of treatment**	-
Yes	20 (23.3)
No	66 (76.7)
**Vascularity**	-
Pink	28 (32.6)
Red	45 (52.3)
Purple	13 (15.1)
**Pigmentation**	-
Hypopigmentation	7 (8.1)
Mixed	37 (43)
Hyperpigmentation	42 (48.8)
**Pliability**	-
Supple	1 (1.2)
Yielding	10 (11.6)
Firm	64 (74.4)
Contracture	4 (4.7)
Rope	7 (8.1)
**Height**	-
< 2 mm	16 (18.6)
2-5 mm	31 (36)
> 5 mm	39 (45.3)
**Total**	86 (100)

**Table 3.** Comparison of Treatment Results in Samples According to Gender, Age Group, Age of Sample at Onset of Scars and Average Scar Duration

Variables	Poor, No. (%)	Moderate, No. (%)	Good, No. (%)	Excellent, No. (%)	P Value
**Gender**	-	-	-	-	0.04
Male	0	0	12.1 (4)	87.9 (29)	-
Female	7.5 (4)	11.3 (6)	17 (9)	64.2 (34)	-
**Age group**	-	-	-	-	< 0.001
≤ 10	42.9 (3)	14.3 (1)	14.3 (1)	28.6 (2)	-
11-20	0	0	0	100 (17)	-
21-40	2.1 (1)	6.2 (3)	18.8 (9)	72.9 (35)	-
41-60	0	40 (2)	0	60 (3)	-
≥ 61	0	0	33.3 (3)	66.7 (6)	-
**Age of sample at onset of scars**	-	-	-	-	< 0.001
≤ 10	42.9 (3)	14.3 (1)	14.3 (1)	28.6 (2)	-
11-20	0	0	0	100 (17)	-
21-40	2.1 (1)	6.2 (3)	18.8 (9)	72.9 (35)	-
41-60	0	40 (2)	0	60 (3)	-
≥ 61	0	0	33.3 (3)	66.7 (6)	-
**Average scar duration**	-	-	-	-	0.1
≤ 1	0	0	0	100 (9)	-
2	7.1 (3)	7.1 (3)	7.1 (3)	78.6 (33)	-
3	0	20 (2)	10 (1)	70 (7)	-
4-5	0	0	0	100 (2)	-
6-9	0	0	40 (4)	60 (6)	-
≥ 10	7.7 (1)	7.7 (1)	38.5 (5)	46.2 (6)	-

**Table 4.** Comparison of Treatment Results of Samples According to Member of Overtaken, Cause of Scarring and History of Treatment

Variables	Poor, No. (%)	Moderate, No. (%)	Good, No. (%)	Excellent, No. (%)	P Value
**Member of overtaken**	-	-	-	-	< 0.001
Chest	0	0	22.2 (2)	77.8 (7)	-
Epigastria and Abdomen	0	0	16.7 (1)	83.3 (5)	-
Hand	7.1 (1)	21.4 (3)	21.4 (3)	50 (7)	-
Breast	0	0	0	100(7)	-
Neck	0	0	0	100 (1)	-
Leg	42.9 (3)	14.3 (1)	0	42.9 (3)	-
Ear	0	100 (2)	0	0	-
Shoulder	0	0	45.5 (5)	54.5 (6)	-
**Cause of scarring**	-	-	-	-	0.1
Deltoid	0	0	0	100 (4)	-
Arm	0	0	8 (2)	92 (23)	-
Surgery	0	14.3 (2)	21.4 (3)	64.3 (9)	-
Acne	0	0	25 (5)	75 (15)	-
Trauma and Rupture	8.8 (3)	2.9 (1)	2.9 (1)	85.3 (29)	-
Folliculitis	0	0	0	100(2)	-
Burn	6.2 (1)	18.8 (3)	25 (4)	50 (8)	-
**History of treatment**	-	-	-	-	0.3
Yes	0	10 (2)	25 (5)	65 (13)	-
No	6.1 (4)	6.1 (4)	12.1 (8)	75.8 (50)	-

**Table 5.** Comparison of Treatment Results of Samples According to Vascularity, Pigmentation, Pliability and Height

Variables	Poor, No. (%)	Moderate, No. (%)	Good, No. (%)	Excellent, No. (%)	P Value
**Vascularity**	-	-	-	-	0.007
Pink	0	0	3.6 (1)	96.4 (27)	-
Red	6.7 (3)	13.3 (6)	15.6 (7)	64.4 (29)	-
Purple	7.7 (1)	0	38.5 (5)	53.8 (7)	-
**Pigmentation**	-	-	-	-	0.06
Hypopigmentation	0	0	0	100 (7)	-
Mixed	2.7 (1)	5.4 (2)	5.4 (2)	86.5 (32)	-
Hyperpigmentation	7.1 (3)	9.5 (4)	26.2 (11)	57.1 (24)	-
**Pliability**	-	-	-	-	< 0.001
Supple	0	0	0	100 (1)	-
Yielding	0	0	0	100 (10)	-
Firm	1.6 (1)	4.7 (3)	15.6 (10)	78.1 (50)	-
Contracture	50 (2)	0	0	50 (2)	-
Rope	14.3 (1)	42.9 (3)	42.9 (3)	0	-
**Height**	-	-	-	-	< 0.001
< 2MM	0	0	0	100 (16)	-
2-5MM	0	0	3.2 (1)	96.8 (30)	-
> 5MM	10.3 (4)	15.4 (6)	30.8 (12)	43.6 (17)	-

## 5. Discussion

In this clinical trial, in which 86 cases with keloid scars were treated with IPL plus corticosteroid intralesional injection, level of clinical improvement, color improvement and scar height was 89.1, 88.8 and 89.1% respectively. Moreover, 73.2% of cases had excellent reactions to treatment and 72.1% had full (100%) recovery. In his study, Kontoes et al. (2003) treated patients with hyperpigmentation hypertrophic scars using IPL plus intralesional injection. It was shown that improvement level in most patients was more than 75% and a decrease of more than 50% was observed in lesion size. This study matches the findings of the present study (9). In a study by Erol et al. (1) in 2008, 109 patients with hypertrophic scars were treated using IPL. In this study, excellent recovery was seen in 31.2% of cases, average improvement in 34% and poor improvement in 9.1%; percentage of cases belonging to each category was less than that of the present study.

Better therapeutic results in the present study can be attributed to the combination of corticosteroid intralesional injection and IPL (1). Using IPL, Han et al. (2007) treated 22 patients with keloid and hypertrophic scars resulting from surgery; all patients achieved clinical improvement which was better than the results of the present study (64.3%) (7). Myers et al. (2005) reported that IPL recovery level was 55% in 107 patients with various skin disorders, which is significantly less than percentage of full recovery in the present study (72.1 %). This difference can be attributed to the diversity of scars and lack of using corticosteroid intralesional injection plus IPL (12). In this study, average number of interventions in 62 patients who achieved complete recovery with IPL was 4.4 ± 1.8. In a study by Han et al. 22 patients with hypertrophic keloid scars resulted from surgery were treated using IPL without injection; on average, 3.5 treatment sessions was reported for clinical improvement of all patients (7). Also Kontoes et al. who treated 19 patients using IPL plus intralesional injection reported that on average 2.97 treatment sessions caused improvement by more than 75% (9). A small sample size could be the reason for the diversity in the results of the reported studies. In this study, a significant difference was observed between the status of response to treatment and the location of keloid scars; excellent response to treatment was less on the ears than other parts of the body. However, in a study by Layton et al. response to treatment was less on the chest than other parts of the body (18). These differences can be due to factors including cause of scar and small sample size. In the present research, level of full improvement varied in terms of causes of scars with the highest improvement level being found in scares caused by trauma and rupture (85.3%) and acne (75%) and the lowest improvement level being found in scares caused by burns (50%). No similar study was found in this regard.

In this study, complications of IPL plus corticosteroid intralesional injection included telangiectasia in 1 case, hyperpigmentation in 7 cases and atrophy in 2 cases. In a study by Erol et al. 109 patients with 12 hypertrophic scars were treated using IPL without injection. In this study, 3 cases of purpura and 1 case of hyperpigmentation were reported. In our study, however, no case of purpura was observed (1). In another study (Kontoes et al.), blister and crust were observed following treatment with IPL plus injection, while no such complications were observed in our study (9). In a study carried out by Manuskiatti et al. in 2002, complications of corticosteroid intralesional injection included hypopigmentation, atrophy and telangiectasia in 50% of treated lesions (19). Myers et al. used IPL in treating skin disorders in 107 patients, 6 of whom experienced complications: blister (1 case), edema (2 cases), vesicol (1 case) and erythema (2 cases). Level of complications was less than that found in our study and type of complications was different from our study (12). In our study, 88.8% of participants were satisfied with IPL; no similar study was found in this regard. The strong point of the present study is that it was carried out in Iran for the first time and that the treatment with IPL was shown to be acceptable.

One of limitations of the present study was its small sample size in terms of place, cause and clinical status of scars; thus, suitable and definite conclusions were impossible regarding the comparison of treatment results according to features of these variables. Another limitation of this study was the absence of a control group. This study has shown that making use of a combined method (injection of corticosteroids in the lesion + IPL) is effective and desirable for improving and treating keloid and hypertrophic scars. It has also been shown that this type of treatment has no significant side effects and results high patient satisfaction and acceptance. IPL is recommended for treating colloid and hypertrophic scars with the height of less than 2 mm and 2-5 mm. Before obtaining more definite results for future studies, a treatment strategy must be selected based on cause, place and vascularity of scars, patients’ viewpoints and their economic conditions. It has also been suggested that further research should be carried out on the efficacy of concentration and dose of corticosteroid plus IPL on height of colloid scars.
